# The Role of the Gut Microbiome in Non-Hodgkin Lymphoma (NHL): A Focus on Diffuse Large B-Cell Lymphoma, Follicular Lymphoma, Cutaneous T-Cell Lymphoma, and NK/T-Cell Lymphoma

**DOI:** 10.3390/cancers17101709

**Published:** 2025-05-20

**Authors:** Magdalena Łyko, Joanna Maj, Alina Jankowska-Konsur

**Affiliations:** 1 Clinical Department of Oncodermatology, University Centre of General Dermatology and Oncodermatology, Wroclaw Medical University, 50-556 Wroclaw, Poland; alina.jankowska-konsur@umw.edu.pl; 2 Clinical Department of General Dermatology, University Centre of General Dermatology and Oncodermatology, Wroclaw Medical University, 50-556 Wroclaw, Poland; joanna.maj@umw.edu.pl

**Keywords:** non-Hodgkin lymphoma, DLBCL, FL, MALT, NK/T-cell lymphoma, CTCL, gut microbiome, dysbiosis, pretreatment, treatment

## Abstract

Non-Hodgkin lymphomas (NHLs) encompass a diverse group of neoplasms arising from the clonal proliferation of B-cell progenitors, T-cell progenitors, mature B-cells, mature T-cells, and natural killer (NK) cells. These malignancies account for over 90% of lymphoid neoplasms. The link between the gut microbiome and neoplasms has been extensively studied in recent years. Growing evidence suggests that the gut microbiome may be involved not only in the development of the disease, but also in modulating the efficacy of implemented therapies. In this review, we summarize the current knowledge on the potential involvement of the gut microbiome in the development of diffuse large B-cell lymphoma (DLBCL), follicular lymphoma (FL), mucosa-associated lymphoid tissue (MALT) lymphoma, and NK/T-cell lymphoma, including cutaneous T-cell lymphoma (CTCL). Moreover, we discuss the relationship between gut microbiome changes before and after treatment and their association with treatment outcomes, focusing on chemotherapy and CAR T-cell therapy.

## Simple Summary

The development and treatment of certain types of lymphomas, including diffuse large B-cell lymphoma, follicular lymphoma, mucosa-associated lymphoid tissue lymphoma, and NK/T-cell lymphoma, may be influenced by the gut microbiome. Recent research suggests that changes in gut bacteria may play a role in both the onset of these diseases and the response to treatments, such as chemotherapy and CAR T-cell therapy. This review aims to summarize the current knowledge on the connection between the gut microbiome and lymphoma, exploring how gut bacteria may affect disease progression and treatment outcomes. Understanding this relationship could help improve therapeutic strategies and lead to more personalized approaches to lymphoma treatment.

## 1. Introduction

The human microbiota describes microorganisms, such as bacteria, viruses, fungi, and arthropods, that inhabit various areas of the human body, including the gastrointestinal tract, oral cavity, skin, and respiratory tract [1,2]. The gastrointestinal tract is one of the largest habitats for diverse microorganisms. Therefore, microbiota impact various physiological functions, such as host metabolism and immune and inflammatory responses [3,4,5]. Among these, the GI tract harbors the most diverse microbial community, influencing host metabolism, immunity, and inflammatory responses [3,4,5]. An imbalance in gut microbiota (dysbiosis) has been implicated in neurologic, psychiatric, respiratory, metabolic, and dermatologic disorders, as well as in malignancies, including non-Hodgkin lymphomas (NHLs) [6,7,8,9,10,11].

This review explores the role of gut microbiota in hematologic malignancies, with a focus on NHLs. We discuss gut dysbiosis in lymphoma development and therapy, microbiota changes across different life stages, and the potential influence of microbiome composition on NHL prognosis.

## 2. Gastrointestinal Microbiota in Health

The composition of gut microbiota is unique to each person and changes during their life [6]. The gut microbiota is influenced by numerous endogenous and exogenous factors. From the very first moments of life, the delivery mode and vertical transmission of microbes shape intestinal microbiota [12,13,14]. In the next stages of life, the composition and diversity of the microbiome change during the colonization process. Moreover, host-associated factors, such as genetics and the immune system response, affect microbial communities [15,16]. Diet, antibiotics, and other drug use, as well as infections, circadian rhythm, and exposure to environmental factors, likewise impact intestinal microbiota [17,18,19]. The best-studied component of the gut microbiome is bacteria. Therefore, most studies focus on alterations in bacterial communities. However, in recent years, scientists have also extended their knowledge on the viral and fungal parts of the intestinal microbiome. Later in this section, we summarize the understanding of a healthy gut microbiome by focusing on the bacterial compartment.

In newborns, the composition of the microbiota, including the intestinal microbes, is unstable and changes dynamically. Bäckhed et al. [14] investigated how the gut microbiota changes due to delivery mode in childbirth and nutrition during the first year of life. Their study showed that the cessation of breastfeeding determined the maturation of the gut microbiota. During this phase of life, the domination of *Bifidobacteria* and *Lactobacilli* was observed in breastfed infants in the first year of life, while in those not fed with breastmilk, *Roseburia*, *Clostridium*, and *Anaerostipes* were more prevalent. Stewart et al. provided evidence that the promotion of gut maturation and defense mechanisms in premature infants is linked to an increase in the consumption of prebiotic oligosaccharides and the proliferation of advantageous microbial strains, such as *Bifidobacteria* [20].

In early childhood, a slowdown in gut microbial expansion is observed. However, gut microbiome diversity is still significantly lower than in adults. In children aged 1 to 5, Cheng et al. [21] reported child-like abundance levels of *Actinobacteria*, *Bacilli*, and *Clostridium* clusters I and IV. However, adult-type levels of *Clostridium* cluster XIVa and *Proteobacteria* were noticed. Moreover, during the 5-year observation time, an increase in the stability or abundance of Bacteroidetes, Clostridium cluster III, and Clostridium cluster XI was reported, which suggests changes in the microbiome toward adult-type profiles. What is more, butyrate-producing bacteria increased gradually during childhood [21].

In adults, the gut microbiota is stable compared to infants. Anaerobic bacteria, including Bifidobacterium, Clostridium, and Bacteroides, gradually replace facultative anaerobic bacteria, such as *Enterobacteriaceae. Bacteroidetes* and *Firmicutes* constitute the most common phyla. In addition, *Actinobacteria*, *Proteobacteria*, and *Verrucomicrobia* are present in the adult microbiome. In addition, *Methanobrevibacter smithii*, archaea that are able to produce methane, yeasts, and phages, can be found in the gut of healthy individuals [22,23]. With aging, the composition of the microbiome changes, becoming less diverse and unstable compared to younger adults [24,25].

However, it is important to emphasize that the gut microbiome is not only made up of bacteria, but also fungi, viruses, and protozoa. As there are currently no reports regarding the influence of the mycobiome and virome on the pathogenesis and outcome of NHL, we will not expand on this topic in this review.

## 3. Gut Microbiome Disbalance and Lymphoma Development

The investigation of the entire gut microbiome and its significance in the pathogenesis and/or treatment of lymphomas is an emerging research interest. Gut microbial dysbiosis is defined as the presence of detrimental species or a lack of beneficial microbes. In the state of physiology, crosstalk between the host immune system and gut microbiome shapes the microbial community and regulates the immunological response to microorganisms present in the intestinal tract [4]. The theory about host–bacteria interactions and how they influence each other’s genome dates back to the previous century. Eukaryotic cells are hypothesized to have arisen from the fusion of two independent ancestral cells, a host cell and an α-proteobacterium, the latter serving as the progenitor of mitochondria, each contributing to protein synthesis and resulting in the development of distinct cytoplasmic and mitochondrial translation systems [26]. The advancement in the development and distinctiveness of bacteria from other prokaryotes was highlighted in 1990, when the new division into the domains archaea, bacteria, and Eucarya was proposed by Carl Woese and colleagues [27].

**Figure 1:**
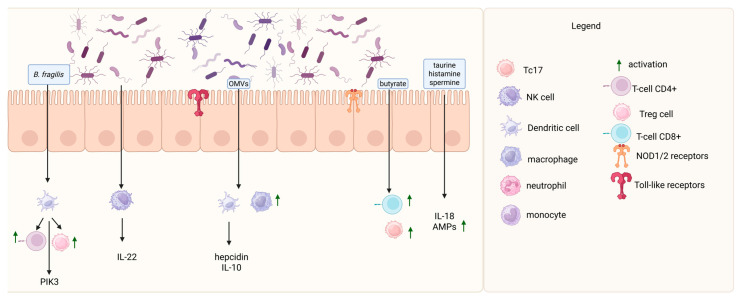
The intricate bidirectional interactions between the gut microbiota and the host immune system. Regarding the *innate immune system,* the gut microbiota modulates innate immunity through several molecular and cellular mechanisms: *Akkermansia muciniphila* can influence the secretion of antimicrobial peptides (AMPs) by intestinal epithelial cells. Pattern recognition receptors (PRRs), such as Toll-like receptors (TLRs) and NOD-like receptors (NLRs), are activated by microbial-derived ligands. These interactions initiate inflammatory responses by upregulating immune gene transcription. *Bacteroides fragilis* engages TLR1/TLR2 heterodimers and Dectin-1, stimulating the PI3K signaling pathway and promoting anti-inflammatory gene expression. NLRs, including NLRP6 inflammasome, are activated by microbial metabolites, such as taurine, histamine, and spermine, leading to IL-18 production and AMP secretion, thus shaping intestinal microbiota composition. Outer membrane vesicles (OMVs) from *Bacteroides* stimulate IL-10 production by dendritic cells (DCs) and enhance macrophage phagocytic activity. Natural killer (NK) cells express RORγt and IL-22 in response to commensal microbiota-derived cues. Concerning the *adaptive immune system,* the adaptive immune compartment is also tightly regulated by gut microbiota-derived signals: Foxp3⁺ regulatory T-cells (Tregs) promote B-cell maturation and secretory IgA (sIgA) production, contributing to microbiota homeostasis and immune tolerance. CD8⁺ cytotoxic T lymphocytes (CTLs) and Tc17 cells are activated by microbial metabolites, such as butyrate, which inhibits histone deacetylases (HDACs), thereby enhancing IFN-γ and granzyme B expression. T helper 17 (Th17) cells are induced by the adhesion of segmented filamentous bacteria (SFB) to intestinal epithelial cells (IECs), contributing to mucosal immunity. T follicular helper (Tfh) cells, modulated by microbiota, are essential for plasma cell and memory B-cell differentiation. Short-chain fatty acids (SCFAs) regulate both the size and suppressive function of the Treg pool, linking microbial metabolism to immune modulation. This diagram underscores the dynamic crosstalk between host immunity and microbial ecology, essential for maintaining intestinal homeostasis. Created with BioRender.com.

Figure 1 presents interactions between the gut microbiota and the host immune system.

The gut microbiome can be involved in lymphomagenesis in a few different ways. First of all, bacteria can stimulate immune cells through various mechanisms via pattern recognition receptors (PRRs) and molecular mimicry, both of which influence the tumor microenvironment by promoting chronic immune activation, inflammation, and immune cell dysregulation. These processes can lead to genetic instability, impaired apoptosis, and uncontrolled proliferation of lymphocytes, creating conditions favorable for lymphomagenesis. Additionally, the dysregulation of the NF-κB pathway caused by gut microbiome alterations further links chronic inflammation to tumorigenesis, contributing to the pro-tumorigenic microenvironment [28].

PRRs expressed on various immune cells are key components of innate immunity. They play a pivotal role in sensing microbial components and regulating immune responses. PRRs, such as Toll-like receptors (TLRs) and nucleotide-binding oligomerization domain-containing proteins (NOD), recognize conserved microbial ligands, including lipopolysaccharides (LPS) and peptidoglycans. In addition to their established roles in host defense and hematopoiesis, PRRs may contribute to lymphomagenesis by driving chronic immune activation, disrupting hematopoietic stem cell regulation, and influencing immune cell maturation. These receptors detect microorganisms through highly conserved molecular patterns, such as bacterial peptidoglycans, flagellin, lipoproteins, and microbial metabolites. Upon activation, different PRRs initiate the expression of antimicrobial peptides (AMPs), promote mucin production, and stimulate the secretion of proinflammatory cytokines. These immune processes can profoundly influence the tumor microenvironment, potentially contributing to tumorigenesis [29,30,31,32,33].

Molecular mimicry is a phenomenon whereby structural or antigenic similarities between microbial and host proteins lead to cross-reactive immune responses. This mechanism, widely implicated in autoimmune and inflammatory diseases, may also contribute to lymphomagenesis through aberrant immune activation. The gut microbiota, particularly through antigenic mimicry, can influence the maturation and activation of immune cells. For instance, microbial peptides resembling host antigens can stimulate antigen-specific T-cell responses. In the context of lymphomagenesis, molecular mimicry may foster chronic immune stimulation and immune cell dysregulation, conditions favorable for lymphocyte transformation. Chronic activation and antigen-specific T-cell responses may indirectly contribute to genetic instability, impaired apoptosis, or unchecked proliferation in lymphocytes, all key features of lymphomagenesis. However, this mechanism can also be involved in anti-tumor mechanisms. Such findings suggest that antigen-specific immune responses induced by molecular mimicry could play a dual role, potentially enhancing anti-tumor immunity in some cases, while promoting chronic immune activation and lymphomagenesis in others [28].

Natural killer (NK) and natural killer T (NKT) cells are a link between innate and adaptive immunity. Moreover, they play an important role in antiviral and antibacterial immunity. With regard to adaptive immunity, the microbiota balance is maintained through the secretion of anti-inflammatory cytokines, the production of secretory IgA (sIgA) mediated by T follicular helper (TFH) cells, and the activation of invariant natural killer T (iNKT) cells mediated by CD1d. A disturbance in microbial communities results in dysregulation of the previously mentioned cytokines’ secretion and, thus, inflammation [34].

Nuclear factor-κB (NF-κB) plays a crucial role in linking inflammation and cancer. Microorganisms and their products are recognized by PRRs that activate the NF-κB pathway and cause inflammation. This recognition process not only influences tumorigenesis, metastasis, and the efficacy and adverse effects of cancer therapy, but also has implications for the overall response to these conditions. In regard to the normal inflammatory response, NF-κB promotes immune cell infiltration through the upregulation of adhesion molecules, chemokines, and vasomodulating factors. However, these same factors are exploited by tumor cells in the inflammatory microenvironment of cancer to facilitate migration and metastasis [35]. Gut microbiome alterations may lead to the increased secretion of pro-inflammatory cytokines, such as TNF, IL-1, and IL-6, resulting in the activation of the NF-κB pathway via appropriate receptors [36]. Chronic inflammation causes prolonged activation of the NF-κB pathway, leading to the creation of a pro-tumorigenic microenvironment [37]. NF-κB dysregulation is commonly observed in various types of human cancer. Tumor cells with constant NF-κB activation often exhibit increased resistance to chemotherapy [35].

As mentioned above, alterations in the gut microbiota may influence the immune system, causing chronic inflammation, which may disturb the balance between cell proliferation and programmed cell death [38,39,40,41]. The abovementioned dysregulation contributes to gene mutation and lymphomagenesis.

The first study showing a relationship between the gut microbiota and lymphoma occurrence was published in 2013 by Yamamoto et al. [42]. Ataxia–telangiectasia (AT) is one of the diseases associated with an increased propensity for lymphomas. This heterogeneous autosomal recessive disorder is associated with pathogenic variants in the ataxia–telangiectasia mutated (ATM) gene. Due to the abovementioned mutation, a defect in DNA repair is observed [43]. Yamamoto et al. [42] investigated the role of the gut microbiota using an ataxia–telangiectasia mouse model to study the penetrance of lymphoma. The results showed that the intestinal microbiota is an essential factor associated with disease penetrance and latency, life length, molecular oxidative stress, and systemic leukocyte genotoxicity. Moreover, the short-term administration of *Lactobacillus johnsonii* in a restricted-microbiota animal model presented reduced systemic inflammation and genotoxicity, resulting in a possible reduction in lymphoma risk, especially in predisposed individuals [42].

## 4. The Role of the Microbiome in B-Cell Lymphomas

B-cell lymphomas represent over 80% of NHLs. The most common subtypes are diffuse large B-cell lymphoma (DLBCL) and follicular lymphoma (FL), which together constitute approximately 55% of all lymphomas [44].

### 4.1. The Role of the Gut Microbiome in DLBCL

Diefenbach et al. [45] analyzed the gut microbiota in a heterogeneous group of NHL patients. The study included a total of 51 subjects diagnosed with DLBCL (*n* = 8), FL (*n* = 13), marginal zone lymphoma (*n* = 5), mantle cell lymphoma (*n* = 3), and T-cell lymphoma. Fifty-one pretreatment fecal samples from the NHL patients were compared with fifty-eight samples from age-matched healthy controls. The study investigated whether the gut microbiome was altered before treatment in the NHL patients in comparison to healthy controls, the differences in the gut microbiome between indolent and aggressive NHL patients before treatment, and the role of the gut microbiota in the treatment response. Firstly, they observed an increase in *Bacteroidetes,* with a simultaneous decrease in *Firmicutes* in NHL patients in comparison to the healthy controls, which proves that the microbiota was altered before treatment. Moreover, the authors examined differences in the gut microbiome between aggressive (DLBCL; *n* = 8) and indolent (MZL; *n* = 8 and FL; *n* = 10) NHL patients before treatment. Aggressive NHL patients were characterized by reduced diversity compared with the indolent types. Further, the investigators studied outcomes in patients receiving immunochemotherapy. Fourteen patients received subsequent treatment, of which eight responded completely or partially to the treatment, while no response to treatment was observed in six patients. In the responders to treatment, the gut microbiome was characterized by higher pretreatment diversity. Moreover, a higher relative abundance of *Dorea formicigenerans* and *Faecalibacterium prausnitzii* was observed in the responders compared to the non-responders. The results suggested an association between the pretreatment microbiome and immunochemotherapy treatment outcomes [45] (Table 1).

Yuan et al. [46] investigated the microbiome composition in fecal samples from 25 untreated DLBCL patients and 26 healthy volunteers. Their study demonstrated a remarkable change in the gut microbiome from the phylum to the family level. The abundance of *Proteobacteria*, *Gammaproteobacteria*, *Enterobacteriales*, and *Enterobacteriaceae* was significantly higher in DLBCL patients than the controls. Moreover, *Escherichia–Shigella*, *Enterococcus*, *Veillonella*, and *Prevotella-2* were substantially more abundant in the DLBCL group. Furthermore, a lower abundance of *Allisonella*, *Lachnospira*, and *Roseburiain* was observed in DLBCL patients. Among bacteria species, an increased abundance of *Escherichia coli* and *Clostridium butyricum* was noticed, whereas the abundance of *Bacteroides fragilis* and *Lactococcus garvieae* was decreased in the DLBCL group. The abovementioned study did not show any differences in terms of the alpha diversity. However, altered microbial composition regarding beta diversity was identified [46] (Table 1).

Schmiester et al.’s [47] study included twelve patients diagnosed with B-cell NHLs (ten DLBCL, one primary mediastinal large B-cell lymphoma, and one high-grade follicular lymphoma) and ten individuals in the control group. They collected from 2 to 11 fecal samples longitudinally before and after the treatment during the follow-up period. This study revealed an increase in *Lachnospiraceae*, showing a taxonomic relationship at the phylum and genus level, compromising *Firmicutes* and *Roseburia*. According to the alpha and beta diversity, no differences were observed in comparison to the healthy controls. However, a significant progressive loss of phenotypic alpha diversity during chemoimmunotherapy was reported. Afterwards, restoration of the diversity during the immunotherapy and follow-up period was observed [47] (Table 1).

Lin et al. [48] presented the gut microbiota of untreated DLBCL patients. The study population consisted of 35 patients and 20 controls. They observed a higher relative abundance of *p_Proteobacteria*, *c_Gammaproteobacteria*, *o_Enterobacteriales*, *f_Enterobacteriaceae*, and *g_ Escherichia–Shigella* in the patients. In the control group, the relative abundance of *p_Bacteroidetes* was lower compared to the DLBCL patients. The remaining findings are presented in Table 1. Furthermore, the authors analyzed the gut microbiome according to the patient’s disease characteristics and immune status. A positive correlation between *p_Firmicutes* and absolute lymphocyte values was observed. Additionally, *g: Prevotella_2* and *s:un_g_Prevotella_2* were negatively correlated with absolute lymphocyte values and T-cell and CD4 cell counts. Moreover, *g_Pyramidobacter*, *s_un_g_Pyramidobacter*, and *f_Peptostreptococcaceae* were negatively correlated with IgA levels [48] (Table 1).

As there is growing evidence of the causal impact of the gut microbiota on lymphoma development, several Mendelian randomization studies have been conducted in the past two years to deepen the understanding of the abovementioned relationship [54,55,56]. Mendelian randomization is a rigorous approach that leverages extensive data from genome-wide association studies (GWAS) to explore genetic associations. The primary advantage of this method is its ability to significantly reduce the influence of confounding factors, such as environmental variables, on the outcomes. MR analysis employs single nucleotide polymorphisms (SNPs) obtained from independent GWAS as instrumental variables (IVs). These SNPs are combined with relevant health outcome data, enabling the estimation of causal relationships within a cohesive framework. Furthermore, this technique allows for the differentiation between causal and non-causal associations, using cross-sectional data.

The role of the tumor microenvironment in cancer development has been emphasized for many years; therefore, in addition to the composition of the microbiome, Jiang et al. aimed to identify which cytokines might be involved in the pathogenesis of DLBCL [54]. Gut microbiota data were sourced from the MiBioGen consortium, comprising 211 microbiota taxa across 18,340 individuals. Genome-wide association study (GWAS) meta-analyses provided data for 47 inflammatory cytokines. Data on 1010 DLBCL cases and 287,137 controls were obtained from the FinnGen consortium. This study provided strong evidence for the causal role of the gut microbiome, specifically the genus *Ruminococcaceae UCG-002*, in the development of DLBCL, with the inflammatory cytokine, MIG, acting as a significant mediator in this relationship. Moreover, the genus *Coprobacter* was a risk factor for DLBCL. The protective effect of the genera *Alistipes* and *Turicibacter* was emphasized in the study. The disease itself influences the gut microbiome, leading to alterations in the abundance of 20 microbial taxa [54].

Another Mendelian randomization study conducted by Li et al. [55] identified five bacterial taxa that had a causal relationship with DLBCL. Scientists identified three taxa, namely the genus *Bilophila*, the family *Desulfovibrionaceae*, and the genus *Coprobacter*, which were associated with a higher risk of DLBCL development. Meanwhile, the genus *Ruminococcaceae* UCG011 and the genus *Alistipes* were negatively correlated with the risk of developing DLBCL [55].

Recently, another large study on the causal role of the gut microbiome and different types of lymphoma was published. The authors identified substantial causal relationships between gut microbiota and lymphoma, contributing valuable insights into the prevention of lymphoma and the development of microbiota-based management strategies. Liang et al. [56] observed a positive correlation between the presence of the genus *Ruminococcaceae UCG002* and the genus *Coprobacter* and the risk of DLBCL. Similarly to Jiang et al. [54], the genera *Alistipes* and *Turicibacter* were associated with a lower risk of DLBCL development.

Qian et al. [57] demonstrated the causal impact of microbiota and metabolites on DLBCL using Mendelian randomization methods. They provided novel insights into the potential targeting of specific microbiota or metabolites for the prevention, diagnosis, and treatment of DLBCL. Investigators examined 119 microorganisms, 1091 plasma metabolites, and 309 metabolite ratios. The genera of microorganisms associated with increased DLBCL risk were *Terrisporobacter* and *Oscillibacter*, while *Methanobrevibacter*, the *Eubacterium coprostanoligenes* group, and *Slackia* were low-risk flora for the development of DLBCL. In addition to microorganisms, specific metabolites, and their ratios were identified as a risk or protective factor for DLBCL. The authors identified that citrate and the taurine/glutamate ratio have protective properties, while glycosyl-N-tricosanoyl-sphingadienine and the phosphoethanolamine/choline ratio were associated with an increased risk of DLBCL [57].

### 4.2. The Role of the Gut Microbiome in FL

Zeze et al. [51] investigated the microbiome composition in gastrointestinal follicular lymphoma (GI-FL), the most common type of primary extranodal FL. The study included 20 GI-FL patients and 20 controls. In contrast to other studies, the authors decided to use mucosal samples from the second portion of the duodenum. The results revealed a lower alpha diversity, with a significant difference in the microbial composition in the GI-FL group compared to the controls. At the genera level *Sporomusa*, *Rothia*, and *Prevotella* were significantly less abundant in the patient group compared to the controls. Similar conclusions were observed at the family level, where the family *Gemellaceae* was significantly less abundant in the GI-FL group (75). A previously mentioned study by Liang et al. [56] showed a positive correlation between the order Bacillales, the family Bacteroidales S24 7 group, the family XIII, the genus *Eubacterium ventriosum* group, and the genus *Ruminiclostridium* 9, with the risk of developing FL. The family *Peptostreptococcaceae*, the genus *Haemophilus*, and the genus *Ruminococcaceae* NK4A214 group were identified as protective factors [56]. While the study by Li et al. [55] showed that the class *Actinobacteria* and the genus *Catenibacterium* were identified as risk factors for the development of FL. Conversely, the family *Pasteurellaceae*, the order *Pasteurellales*, the genus *Alistipes*, the genus *Haemophilus*, the genus *Slackia*, the family *Peptostreptococcaceae*, the family *Rhodospirillaceae*, and the genus *Ruminococcaceae* demonstrated a reduced risk of FL [55] (Table 1).

Xu et al. [53] analyzed gut microbiota composition in 28 treatment-naïve patients. In contrast to the abovementioned study, the authors identified an overabundance of the *Ruminococcaceae* family and a decreased abundance of the *Coriobacteriaceae* family in FL patients. Moreover, a high level of *Ruminococcus* was recognized as a strong indicator of tumor burden (Table 1).

Based on the above, the findings across different studies remain inconsistent, and several limitations restrict broad conclusions being drawn. Diefenbach et al. [45] and Zeze et al. [51] reported reduced alpha diversity in NHL patients compared to healthy controls, particularly in regard to aggressive subtypes or GI-FL. In contrast, Schmiester et al. [47] and Yuan et al. [46] found no significant differences in alpha diversity between patients and controls. These discrepancies may stem from small sample sizes, heterogeneous patient groups, and variations in sequencing platforms and analysis pipelines used.

### 4.3. Evidence for a Causal Relationship Between Gut Microbiota and B-Cell Lymphomas

While many observational studies have demonstrated alterations in gut microbiota composition in patients with DLBCL and FL, these findings alone cannot confirm a causal relationship due to potential confounding factors and reverse causation. However, Mendelian randomization studies provide robust evidence supporting a causal link between specific bacterial taxa and the risk of developing DLBCL and FL.

MR analyses utilize genetic variants (SNPs) as instrumental variables to infer causality, significantly reducing bias from environmental or behavioral confounders. Several studies [54,55,56,57] have employed this approach to identify microbial genera, such as *Ruminococcaceae UCG-002*, *Coprobacter*, and *Bilophila*, as risk factors for DLBCL. At the same time, *Alistipes*, *Turicibacter*, and *Slackia* appear to play a protective role. Additionally, Jiang et al. [54] demonstrated that the pro-inflammatory cytokine, MIG, mediates the effect of *Ruminococcaceae UCG-002* on lymphoma development, suggesting a mechanistic pathway linking microbiota to host immune modulation.

Similar causal associations were observed for FL. For instance, Liang et al. [56] and Li et al. [55] identified bacterial taxa such as *Haemophilus*, *Peptostreptococcaceae*, and *Ruminococcaceae NK4A214 group* as protective, while others, including Catenibacterium and Bacteroidales S24-7 group, were associated with increased disease risk.

These findings suggest that gut microbiota may not only reflect the disease state, but also actively contribute to lymphomagenesis. The integration of MR data with observational and mechanistic studies supports the hypothesis that microbial dysbiosis plays a causal role in the pathogenesis of B-cell lymphomas. This has profound implications for the development of microbiota-based prevention and therapeutic strategies in the future.

## 5. The Role of the Microbiome in Mucosa-Associated Lymphoid Tissue (MALT) Lymphoma

Mucosa-associated lymphoid tissue (MALT) lymphoma is the most common hematological neoplasm located in the stomach. They constitute between 50–72% of all lymphomas in this localization [58].

The role of *Helicobacter pylori* in the development of MALT lymphoma has been widely studied. Chronic inflammation caused by *H. pylori* has been identified as a crucial factor in lymphomagenesis. Helicobacter pylori infection can induce dysbiosis in both the stomach and intestinal microbiomes. Moreover, virulence proteins, such as vacuolating cytotoxin A (VacA) and cytotoxin-associated gene A (CagA), cause direct damage to the gastric epithelium and trigger microbial imbalance [59].

However, in *H. pylori*-negative MALT lymphoma, the gastric microbiome has been analyzed, as the pathogenesis of this entity remains unclear. Tanaka et al. [52] compared the microbiome of 33 *H. pylori*-negative gastric MALT lymphoma patients and 27 healthy controls, identifying bacteria other than *H. pylori* that may be involved in the pathogenesis of *H. pylori*-negative MALT lymphoma. At the genus level, a higher abundance of Burkholderia and Sphingomonas and a lower abundance of Prevotella and Veillonella were observed in MALT lymphoma patients compared to the controls. Interestingly, at the genus level, Helicobacter was detected in MALT lymphoma patients and controls; thus, the abundance was insignificant in both groups compared with the abundance in data from patients with *H. pylori*-positive samples. Lower alpha diversity was observed in *H. pylori*-negative MALT lymphoma patients and significant beta diversity differences were observed between the MALT group and the controls [52] (Table 1).

## 6. The Role of the Microbiome in NK/T-Cell Lymphomas

NK/T-cell lymphomas are a heterogeneous and aggressive subtype of NHL. Shi et al. [50] sought to identify diagnostic and prognostic biomarkers in a cohort of 30 untreated natural killer/T-cell lymphoma (NKTCL) patients and 20 healthy controls. They noticed an increase in *Veillonella* and *Streptococcus* in the NKTCL group. Moreover, in the control group, an increase in *Faecalibacterium prausnitzii*, *Eubacterium rectale*, and *Bifidobacterium adolescentis* was observed. These microbial changes can serve as a useful biomarker for distinguishing NKTCL patients from the control group, but not from other diseases. Based on the obtained data, scientists have proposed a *Streptococcus parasanguinis–Romboutsia timonensis* index (SRI) to predict the overall survival (OS) and progression-free survival (PFS) in patients. The higher the index, the higher the OS and PFS of the patient [50] (Table 1).

Cutaneous T-cell lymphomas (CTCLs) are a heterogeneous group of neoplasms, with the primary presentation in the skin. The contribution of infectious agents in the etiology and pathogenesis of CTCL has been proposed in the past [60]. In particular, the involvement of Staphylococcus aureus, the human T-lymphotropic virus (HTLV), the Epstein–Barr virus, and human herpes virus-8 was reported [61,62,63]. Most controversies were related to the connection between HTLV infection and CTCL development [64,65,66,67,68]. However, to date, no link between a specific pathogen and CTCL pathogenesis has been identified.

As CTCLs are characterized by primary skin involvement, several studies have investigated the composition and diversity of the skin microbiome in regard to those entities. A summary of the current knowledge on the topic of the skin microbiome in CTCLs was discussed in detail in our last review [69]. Moreover, gut dysbiosis was observed in several inflammatory skin diseases, such as atopic dermatitis, psoriasis, or hidradenitis suppurativa, showing the association of the gut microbiome with the mentioned diseases’ severity [70,71].

Hooper et al. [49] included 38 CTCL patients (27 with mycosis fungoides, 5 with Sézary syndrome, and 6 with non-MF/SS CTCL) and compared them with 13 healthy controls. In the CTCL patients, the gut microbiome was characterized by lower α-diversity, with statistical differences between the advanced CTCL group and the controls. Moreover, a significant decrease was observed in the phylum Actinobacteria, specifically in the classes Coriobacteria and Actinobacteria, the order Coriobacteriales, and the genus Anaerotruncus. In patients with advanced disease, significant *Eggerthellaceae* and *Lactobacillaceae* reductions were reported [49] (Table 1). It should be noted that in this case-control study, the examined population was small, and further multicenter studies are needed to reach further conclusions.

A previously mentioned study by Liang et al. [56] also analyzed the link between the microbiome and mature T/NK-cell lymphomas. The authors observed that the genus *Ruminococcaceae UCG004* was positively correlated with the risk of lymphoma. While the family *Methanobacteriaceae* and the genus *Methanobrevibacter*, the family *Lactobacillaceae* and the genus *Lactobacillus*, the family *Verrucomicrobiaceae* and the genus *Akkermansia*, the genus *Bifidobacterium*, the genus *Eubacterium oxidoreducens* group, the genus *Ruminococcaceae UCG014*, and the genus *Lachnospiraceae UCG001,* are associated with a lower risk of mature T/NK-cell lymphomas [56].

Interestingly, Li et al. [55] did not identify any bacterial taxa that are associated with an increased risk of NK/T-cell lymphoma. Instead, eight bacterial taxa were revealed to have a lower risk of disease, including the genus *Ruminococcus1*, the R.7 group of *Christensenellaceae*, the family *Lactobacillaceae*, the genus *Lactobacillus*, *Ruminococcaceae UCG014*, the order *Methanobacteriales*, the class *Methanobacteria*, and *Lachnospiraceae UCG001.*

As the results are conflicting, further research is needed to determine whether NK/T-cell lymphoma development is indeed microbiome dependent.

Importantly, none of the current studies include interventional data or longitudinal microbiome analyses that would substantiate a causal relationship. Moreover, germ-free animal models, fecal microbiota transplantation, and mechanistic studies, hallmarks of microbiome causality research, are lacking in the context of NK/T-cell lymphomas. Therefore, while associative evidence is mounting, causal inference remains speculative.

Although correlations have been identified between gut and skin microbiota and NK/T-cell lymphomas, robust evidence of a causal relationship is currently insufficient. Future studies should incorporate experimental models, longitudinal cohort data, and interventional designs to elucidate whether the microbiota plays a mechanistic role in lymphomagenesis or merely reflects disease-associated immune dysregulation.

## 7. The Association Between the Gut Microbiome and Anticancer Treatment

Various studies have investigated the relationship between gut microbiota and anticancer treatment. On the one hand, bacteria can influence the efficacy of systemic anticancer therapy. Microorganisms may increase drugs’ cytotoxicity or reduce their side effects. On the other hand, CHTH, immunotherapy, and hormonal therapy alter the composition and diversity of the gut microbiome [72,73]. Moreover, the alterations of gut microbiota before treatment can also be useful in predicting treatment outcomes and the development of complications [73].

So far, research on the gut microbiota–anticancer treatment association has focused on colorectal cancer [74,75,76,77], acute myeloid leukemia [78,79], NHL [80,81], breast cancer [82], lung cancer [83], ovarian cancer [84], hepatocellular carcinoma [85], metastatic melanoma [86,87,88], and metastatic renal cell carcinoma [89]. Here, we discuss data on hematoproliferative disorders, focusing on studies including NHL patients.

### 7.1. The Gut Microbiome Composition and Chemotherapy (CHTH)

Montassier et al. [80], in their first study, included eight patients receiving high-dose CHTH. From each patient, samples were collected before and one week after the bone marrow transplant conditioning CHTH. The abovementioned study revealed a significant reduction in alpha diversity. Moreover, on the phylum level, a decrease in Firmicutes and Actinobacteria was reported, while *Bacteroidetes* and *Proteobacteria* were significantly more abundant in post-chemotherapy samples. The authors reported that at the genus level, the proportion of *Bacteroides* and *Escherichia* significantly increased, and the proportion of *Blautia*, *Faecalibacterium*, and *Roseburia* significantly decreased after chemotherapy.

Another study by Montassier et al. [81] involved 28 NHL patients being investigated to identify the gut microbiome composition before myeloablative conditioning CHTH to identify alterations that may predict the risk of bloodstream infection (BSI). Of the 28 patients, eleven developed BSI caused by *Escherichia coli* (*n* = 4), *Enterococcus* (*n* = 2), and other *Gammaproteobacteria* (*n* = 5). A lower alpha diversity and reduced richness and evenness were observed in patients who developed BSI compared to the non-BSI group. Moreover, a significantly decreased abundance of members of *Actinobacteria (Coriobacteriaceae)*, *Bacteroides (Barnesiellaceae*, *Butyricimonas)*, *Firmicutes (Christensenellaceae*, *Faecalibacterium*, *Oscillospira*, *Christensenella*, *Dehalobacterium)*, *Proteobacteria (Desulfovibrio*, *Sutterella*, *Oxalobacter)*, and a higher abundance of *Erysipelotrichaceae* and *Veillonella* were observed in fecal samples.

Xu et al. [90], in a prospective study, assessed a cohort of 17 untreated DLBCL patients compared to 18 healthy controls. The authors observed an increased abundance of *Proteobacteria* and *Enterobacteriaceae*, specifically *Escherichia coli*. Patients who achieved complete remission had higher levels of *Lactobacillus fermentum*, *Lactobacillus*, and *Lactobacillaceae,* compared to those who did not achieve complete remission. The appearance of *Lactobacillus fermentum* during chemotherapy was associated with tumor reduction and a better clinical response. The distinct gut microbiome profiles between CR and NCR patients indicate that the gut microbiome composition could serve as a predictor of the treatment response. Specifically, the presence of *Lactobacillus* species during treatment might represent a biomarker for better outcomes. Additionally, the study found an overrepresentation of virulence factors and other bacterial families, such as *Veillonellaceae* and *Rikenellaceae*, in patients with different clinical responses, further supporting the potential role of the gut microbiome in modulating treatment effectiveness.

Yoon et al. [91] investigated the relationship between gut microbiota and outcomes in newly diagnosed, treatment-naïve patients with DLBCL undergoing RCHOP chemotherapy. Using 16S rRNA and whole-genome sequencing (WGS) of baseline stool samples, the study revealed that DLBCL patients had significantly lower microbial diversity and an increased abundance of Enterobacteriaceae, including species like *Escherichia coli* and *Klebsiella pneumoniae*, compared to healthy controls. This gut dysbiosis was linked to an increased risk of febrile neutropenia and poorer treatment outcomes. The study found that patients with febrile neutropenia had distinct microbiota profiles, with a predominance of pathogens known to contribute to chemotherapy complications. Elevated levels of inflammatory markers, such as IL-6 and IFN-γ, were also associated with *Enterobacteriaceae* abundance. These findings suggest that gut dysbiosis in DLBCL patients may play a role in promoting inflammation and adverse clinical outcomes. The authors propose that strategies to restore a healthy gut microbiome, including increasing commensal bacteria, such as *Faecalibacterium* and *Bifidobacterium*, may help reduce *Enterobacteriaceae* levels and improve patient outcomes.

### 7.2. The Gut Microbiome Composition and CAR T-Cell Therapy

Chimeric antigen receptor T-cell (CAR T-cell) therapy revolutionized the treatment of multiple hematological malignancies, including B-cell lymphomas. As CAR T-cell therapy is available to patients in clinical settings, patients are under medical supervision. Like any therapy, it is associated with the occurrence of severe adverse events (SAEs). Cytokine release syndrome (CRS) and immune effector cell-associated neurotoxicity syndrome (ICANS) are among the most commonly reported SAEs. Moreover, some patients do not respond to the treatment or relapse after therapy [92,93]. Therefore, there is a need to find predictive factors that indicate those groups of patients and enable the modification of external factors, leading to better therapeutic outcomes. It seems that microbiome alterations may impact the efficacy of this therapy [94,95,96].

The correlation between CD-19 CAR T-cell therapy and the gut microbiome in B-cell lymphoma was analyzed by Smith et al. [97]. In a multicenter study performed in the USA, 137 patients diagnosed with NHL and 91 patients diagnosed with acute lymphoblastic leukemia were included. Here, we will just focus on the NHL patients. Firstly, they retrospectively analyzed the relationship between antibiotic use 4 weeks before the treatment with and the outcome of CAR T-cell therapy. Significantly, exposure to antibiotics was linked to worse overall survival (OS) in patients. However, no such association was observed with progression-free survival (PFS). Researchers also compared whether the use of specific antibiotics is associated with worse OS and PFS. In NHL patients who were exposed to piperacillin–tazobactam, imipenem–cilastatin, and meropenem, significantly shorter OS and PFS were observed. Moreover, they observed that exposure to P-I-M antibiotics within the four weeks preceding CAR T-cell infusion was linked to a reduction in OS, but not in progression-free survival (PFS), among NHL patients, regardless of the specific CAR costimulatory domain utilized.

Hu et al. [98] longitudinally monitored changes in the microbiota throughout the CAR T-cell therapy process, utilizing stool samples obtained before CAR T-cell infusion, during infusion but before the onset of CRS, during active CRS, and up to fourteen days post-infusion. The study group included patients diagnosed with multiple myeloma (MM), NHLs, and acute lymphoblastic leukemia (ALL). However, all the study groups were analyzed separately. Severe CRS episodes were correlated with a reduction in the abundance of Bifidobacteria. The Shannon index, reflecting the alpha diversity, significantly declined following CAR T-cell infusion, concurrent with a notable increase in the abundance of Actinomyces and *Enterococcus* genera. Additionally, patients who achieved a complete response exhibited higher levels of *Prevotella*, *Collinsella*, *Bifidobacterium*, and *Sutterella* spp., compared to those with a partial response.

Stein-Thoeringer et al. [99] investigated whether broad-spectrum antibiotic use prior to CAR T-cell therapy impacts patient outcomes. Their study showed that antibiotic intake before CAR T-cell therapy was associated with poorer outcomes. However, this effect appears to be confounded by a higher pre-treatment tumor burden and systemic inflammation in patients who received high-risk antibiotics. The most important bacterial taxa that determined CAR T-cell responsiveness were *Bacteroides*, *Ruminococcus*, *Eubacterium*, and *Akkermansia.* Moreover, *Akkermansia* was associated with pre-infusion peripheral T-cell levels in these patients, and, based on this multicenter study, the authors identified conserved microbiome signatures across clinical and geographical settings, potentially enabling cross-cohort microbiome-based predictions of outcomes in CAR T-cell immunotherapy.

### 7.3. Antibiotics, Anticancer Treatment, and the Microbiome

For years, there has been an ongoing debate regarding the necessity of prophylactic antibiotic therapy in patients with NHL [100,101]. CAR T-cell therapy and CHTH are linked to complications, such as neutropenia, lymphopenia, and others, which may consequently increase the risk of infections in patients. According to the NCCN clinical guidelines regarding the prevention and treatment of cancer-related infections, routine antibiotic prophylaxis should be assessed individually, with particular consideration given to the underlying malignancy, the degree of neutropenia, the history of infections, exposure to pathogens, treatment with myelosuppressive regimens, and the overall status of the immune function in the patient [102]. Still, in many facilities, broad-spectrum antibiotic prophylaxis is customarily used in clinical practice. While antibiotics provide protection against infections, this approach will disturb the balance of the intestinal microbiota and, thus, may affect anticancer treatment.

Kuczma et al. [103] investigated the impact of antibiotic administration on the therapeutic outcomes of diverse cancer treatment modalities, specifically involving the use of cyclophosphamide (CTX), either as monotherapy or as a host pre-conditioning regimen, for adoptive cell therapy (ACT) in mouse models. Moreover, they investigated whether changes in the gut microbiota through antibiotic exposure influence the efficacy of CD19- CAR T-cell therapy. This investigation revealed that in an animal model of B-cell lymphoma, administering antibiotics as a preventive measure reduced the therapeutic efficacy of CTX against B-cell lymphoma. In the context of ACT, the administration of antibiotics led to a decrease in the effectiveness of tumor-specific CD4+ T-cells in a colorectal tumor model. However, long-term antibiotic exposure did not affect the efficacy of ACT using CD19- CAR T-cells in treating systemic lymphoma. Nonetheless, it did impact the persistence of CAR expression and the duration of B-cell aplasia [103].

However, the study by Prasad et al. [104] showed that patients receiving broad-spectrum antibiotics with extended anaerobic coverage, such as piperacillin–tazobactam and meropenem, exhibited worse survival outcomes following the therapy. The authors investigated the effects of broad-spectrum antibiotic exposure on the gut microbiome and clinical outcomes in patients receiving CD19- CAR T-cell therapy for large B-cell lymphoma. Microbiome disruption was associated with significant alterations in the gut and blood metabolome, including reductions in short-chain fatty acids, indole, and cresol derivatives, and trimethylamine N-oxide, which are critical for immune and metabolic homeostasis [104].

It seems that individualizing antibiotic therapy according to specific risk factors is essential for optimizing treatment outcomes.

When discussing the necessity of antibiotic therapy in patients undergoing CAR T-cell treatment, it is worth mentioning the work of Pernas et al. [105]. They investigated the frequency and severity of infectious complications after CAR T-cell therapy in patients with aggressive B-cell lymphoma, who did not use antibiotic prophylaxis. Among the 137 patients included in the study, 41 patients (30%) experienced a total of 63 infectious events. Infection-related mortality was reported in two patients. The majority of infections occurred during hospitalization for CAR T-cell therapy. The identified independent risk factors for infection included the male gender, prior autologous hematopoietic cell transplantation, three or more previous lines of therapy, and pre-lymphodepletion neutropenia.

## 8. Future Directions

The future of the treatment of NHLs is the development of personalized therapies, including microbiome-targeted therapies. These innovative treatments could offer a minimally toxic alternative for aggressive and treatment-resistant lymphomas, addressing an unmet medical need. As dysbiosis has been linked to increased mortality, gut microbiome restoration seems to be essential to observe satisfactory outcomes. This encompasses auxiliary diagnosis, novel prevention and treatment strategies, and predicting clinical outcomes and treatment-related adverse effects. Given the apparent influence of gut dysbiosis on the occurrence of adverse events, especially regarding CAR T-cell therapy, further studies on microbiome restoration before treatment may improve patient outcomes.

Antibiotic prophylaxis may offer protection against infections; however, its use, especially broad-spectrum antibiotics, should be carefully considered in patients undergoing anticancer therapy, due to potential disruptions to the microbiome, which may negatively influence treatment efficacy. Tailoring antibiotic use based on individual risk factors is crucial for optimizing outcomes. Further studies are needed to fully understand the impact of antibiotics on the microbiome and cancer therapy outcomes.

It is crucial not to overlook other microbiome constituents, such as viruses and fungi. Data on the mycobiome in lymphoma or leukemia are currently lacking, presenting an opportunity for further research. Moreover, characterizing viral communities poses challenges, but holds immense potential for shedding light on the development of NHLs and identifying novel treatment methods, including bacteriophages.

## 9. Conclusions

As presented in this review, the association between the microbiome and NHLs has been extensively studied, revealing disruptions in the gut microbiota among patients prior to treatment initiation, as well as due to the implemented therapy. Moreover, the host–microbiome interaction seems to impact the development of disease, per se.

In summary, ongoing research efforts should focus on elucidating causal links between microbiota alterations and lymphoma development, exploring novel therapeutic modalities, and comprehensively characterizing the microbiome, including the bacterial, viral, and fungal components, to advance personalized therapies in NHL treatment. Moreover, it seems that the modulation of the gut microbiome, leading to maintaining its balance, is important for both prevention and treatment strategies.

## Figures and Tables

**Table 1:** A summary of the studies on the gut microbiome prior to treatment in patients with NHLs.

Authors (Year)	Study Population and Study Type	Sample Type	Method	Observed Changes in NHL Patients
Yuan et al. (2021) [46]	25 untreated DLBCL patients vs. 26 healthy controls; cross-sectional study	Fecal samples	16s rRNA sequencing	↑ abundance of *Proteobacteria*, *Gammaproteobacteria*, *Enterobacteriales*, *Enterobacteriaceae*, *Escherichia–Shigella*, *Enterococcus*, *Veillonella*, and *Prevotella-2* ↓ abundance of *Allisonella*, *Lachnospira*, and *Roseburiain*
Diefenbach et al. (2021) [45]	51 NHL patients vs. 58 healthy controls; cross-sectional study	Fecal samples	16s rRNA sequencing	↑ *Bacteroidetes* with a simultaneous decrease in *Firmicutes* ↑ relative abundance of *Dorea formicigenerans* and *Faecalibacterium prausnitzii* in responders
Schmiester et al. (2022) [47]	10 DLBCL, 1 primary mediastinal large B-cell lymphoma, and 1 high-grade FL vs. 10 controls; cross-sectional study	Fecal samples	single-cell flow cytometry (FCM) and 16s rRNA sequencing	↑ abundance of *Firmicutes*, the family *Lachnospiraceae,* and the genus *Roseburia* ↓ α-diversity during CHTH
Lin et al. (2023) [48]	35 untreated DLBCL patients vs. 20 healthy controls; cross-sectional study	Fecal samples	16s rRNA sequencing	↓ β-diversity *, ↑ abundance of *p_Proteobacteria* *, *p_Verrucomicrobia* *, *p_Synergistetes* *, *g_Escherichia–Shigella* *, *g_Veillonella* *, g_Roseburia *, *g_Lachnoclostridium* *, g_Alistipes * ↓ abundance of p_Bacteroidetes *, g_Bacteroides *, g_Prevotella_9 *, and g_Megamonas *
Hooper et al. (2022) [49]	27 MF, 5 SS, and 6 with non-MF/SS CTCL vs. 13 healthy controls; cross-sectional study	Fecal samples	16s rRNA sequencing	↓ α-diversity
Shi et al. (2022) [50]	30 untreated NKTCL patients vs. 20 healthy controls; cross-sectional study	Fecal samples	shotgun metagenomic sequencing	↑ *Veillonella* and *Streptococcus* in NKTCL group
Zeze et al. (2020) [51]	20 GI-FL patients vs. 20 controls; cross-sectional study	Mucosal biopsy samples from the second portion of the duodenum	16s rRNA sequencing	↓ α-diversity * ↓ abundance of *Sporomusa*, *Rothia*, *Prevotella*, and the family *Gemellacea* *
Tanaka et al. (2021) [52]	33 *Helicobacter pylori*-negative MALT lymphoma patients vs. 27 controls; cross-sectional study	Mucosal biopsy from the gastric body	16s rRNA sequencing	↑ abundance of *Burkholderia* and *Sphingomonas* ↓ abundance of *Prevotella* and *Veillonella* ↓ α-diversity
Xu et al. (2024) [53]	28 treatment-naïve FL patients vs. 18 sex- and age-matched healthy controls; cross-sectional study	Fecal samples	16s rRNA sequencing	↑ abundance of *Ruminococcaceae* ↑ *Ruminococcus* is a strong indicator of tumor burden ↓ abundance of *Coriobacteriaceae*

DLBCL—diffuse large B-cell lymphoma; NHL—non-Hodgkin lymphoma; FL—follicular lymphoma; GI-FL—gastrointestinal follicular lymphoma; MALT—mucosa-associated lymphoid tissue lymphoma; MF—mycosis fungoides; SS—Sezary’s syndrome; CTCL—cutaneous T-cell lymphoma; CHTH—chemotherapy; NKTCL—natural killer/T-cell lymphoma; *—statistically significant difference between the study group and the controls; ↑ higher; ↓ lower.
